# Supplementary figures and images for: Long-Term Outcome and Quality of Life in Patients Treated for Head and Neck Sarcoma during Pediatric Age: Considerations from a Series of 4 Cases
Source: Reports (MDPI). 2023 Mar 17;6(1):16. doi: 10.3390/reports6010016 (PMC12225406; doi:10.3390/reports6010016)

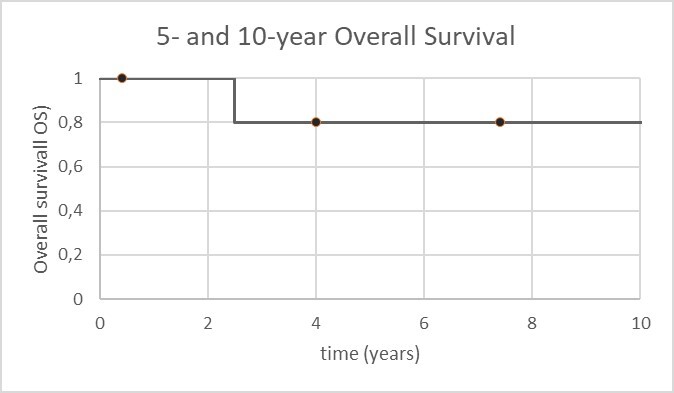

Supplement: Supplementary file 1 [file reports-06-00016-s001.zip › Figure S1.jpg]
